# Late Development of Pancreatitis Following Gunshot Trauma, A Case Report

**Published:** 2012-09-30

**Authors:** S Nikeghbalian, M Akrami, A Fazelzadeh

**Affiliations:** 1 Associate Professor of Surgery, Shiraz University of Medical Sciences, Shiraz, Iran; 2 General Surgeon, Shiraz University of Medical Sciences, Shiraz, Iran; 3 Resident of General Surgery, Shiraz University of Medical sciences, Shiraz, Iran

**Keywords:** Late, Pancreatitis, Gunshot, Trauma

## Abstract

**Background:**

Gunshot trauma to the pancreatic duct mainly have been published from wartime experiences, but bullet injury in these cases has lead to pancreatic duct disruption, not obstruction. We report a case of chronic pancreatitis which is presented 27 years following pancreatic duct obstruction due to bullet injury during wartime, which successfully treated. He was a 45-year-old man came with chronic epigastric abdominal pain. Physical examination was suggestive of pancreatitis and laboratory data confirmed the diagnosis. Imaging studies revealed a metallic object in main pancreatic duct. He carefully treated with pancreatic head resection and pancreaticojejunal anastomosis.

## Introduction

Obstruction of the pancreatic duct may leads to acute or chronic pancreatitis.

This obstruction can be due to stone or tumors, (1) but amazingly, 3 mm caliber bullet can also block pancreatic duct which has a same diameter. (2)

Specific experiences of gunshot trauma to the pancreatic duct mainly have been published from wartime experiences, but bullet injury in these cases has lead to pancreatic duct disruption, not obstruction.

We report a case of chronic pancreatitis which is presented 27 years following pancreatic duct obstruction due to bullet injury during wartime, which successfully treated.

## Case Report

A 45-year-old man came with chronic epigastric abdominal pain of variable severity which usually occurred 30 to 60 minutes after meals. He had nausea frequently. He didn't have Vomiting, chills, fever or jaundice. He had been treated for these symptoms by giving various antibiotics, antiacid and analgesics without lasting relief.

His past history revealed that he had gunshot injury during war time 27 years ago and underwent operation at that time and after that he has been having several episodes of abdominal pain.

On physical examination, the scar of previous laparotomy and the scar of gunshot entrance on left flank were seen.

There was mild tenderness on deep palpation in the epigastrium. No muscle spasm or rebound tenderness was present.

There were no palpable masses. The remainder of the examination was not remarkable.

Laboratory analysis showed hemoglobin of 11/100 ml, white blood count of 7,000/mm^3^, serum amylase of 620 Somogyi units. The AST, ALT, LDH, and alkaline phosphatase levels were normal.

**Fig 1:**
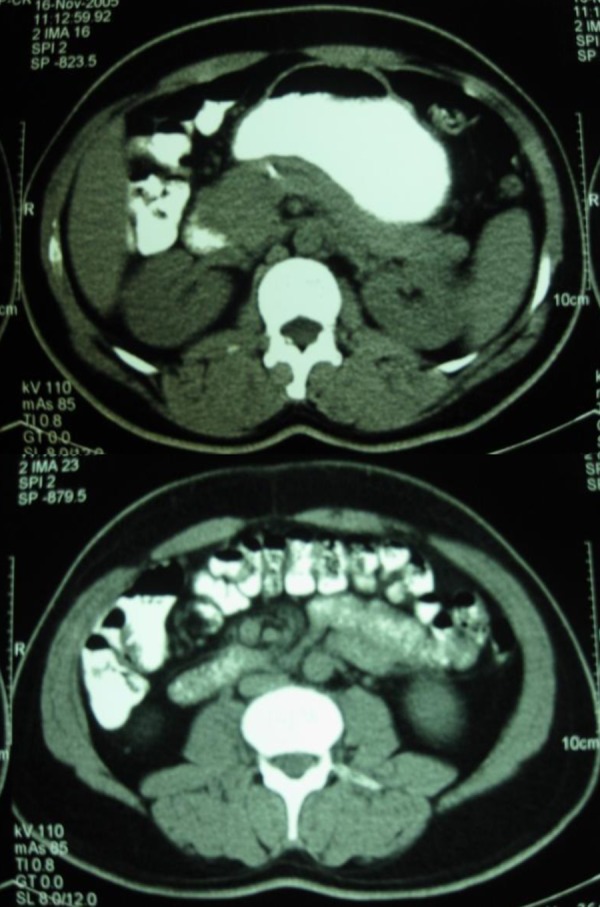
Abdominal CT scan showing pancreatitis (black star) and a metallic lesion in the course of the main pancreatic duct (white arrow)

The computed tomography (CT) scan of the abdomen after intravenous-bolus contrast medium injection showed diffuse enlargement of the pancreas with inhomogeneous staining of its parenchyma and a metallic lesion within pancreas in the course of main pancreatic duct (Figure 1).

**Fig 2:**
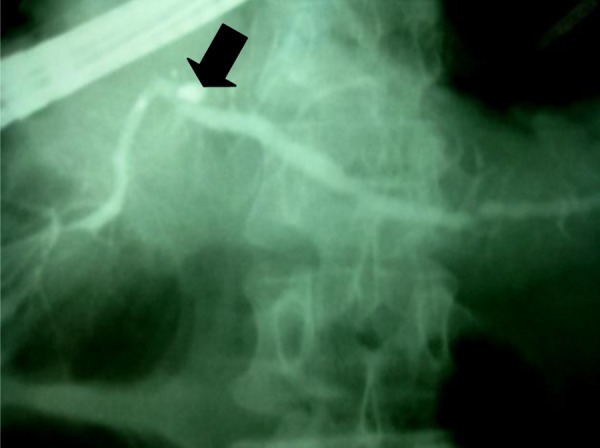
Pancreaticogram demonstrating bullet in the pancreatic duct (black arrow)

Endoscopic retrograde cholangiopancreaticography (ERCP) showed proximal pancreatic duct obstruction due to a bullet in the main pancreatic duct ERCP did not show any dye leakage, due to pancreatic duct disruption (Figure 2).

The exploratory laparotomy confirmed the diagnosis of pancreatitis. After a midline laparotomy, the subtotal resection of the pancreatic head was done and reconstruction was performed with a Roux-en-Y loop of the jejunum with an end-to-side pancreatico-jejunal anastomosis.

The patient was discharged after 10 days in a good condition and he didn't have any complaint in his 6 months follow-ups till now.

## Discussion

The incidence of penetrating injuries in the pancreas has been reported to be 1% and overall morbidity and mortality ranges from 30 to 100%. (3,4)

In 45-80% of pancreatic injuries, damage to surrounding organs also occurs which result in considerable morbidity and mortality. But in the cases with isolated penetrating pancreatic duct injury, spontaneous closure of pancreatic ductal disruption has been reported. (5)

Early diagnosis and adequate treatment for pancreatic trauma are essential for the prevention of complications. (6)

As retroperitoneal lesions do not have any specific symptoms, diagnosis of pancreatic duct injury is difficult even in the presence of penetrating trauma. Enzyme levels can be normal in 30-40% of patients with pancreatic ductal injury therefore elevation of serum amylase is not a specific sign of pancreatic injury. (4) 

Abdominal CT,can detect parenchymal lesions but it is not the procedure of choice in diagnosis of ductal injury. Gougeon *et al*. reported the use of emergency ERCP in the diagnosis of pancreatic injury for the first time in 1976. (7) Now it is known as the gold standard for diagnosis of pancreatic ductal injury. ERCP should be carried out whenever main pancreatic duct injury is suspected. It could provide not only a conclusive diagnosis, but also an effective and safe non-operative treatment tool. (8)

In this case we have a history of gunshot to abdomen 27 years ago during wartime, he underwent emergency laparotomy due to his unstable condition, The diagnosis of pancreatic injury had been even more difficult in that situation because of associated major organ injuries .Therefore the pancreatic duct injury had not been diagnosed . After many years pancreatitis was developed due to obstruction of duct by a bullet, which was diagnosed and treated successfully.

Although ERCP can help in early diagnosis of pancreatic duct injury but it is not available in critical situations like war, and it can't be done for unstable patients.

For early diagnosis and reducing the mortality of these patients, probability of pancreatic injury should keep in mind for every patients with abdominal gunshot trauma. 
